# Multi-omic profiling reveals age-specific blood biomarkers and aging-driven B cell remodeling in osteoarthritis

**DOI:** 10.1097/JS9.0000000000003076

**Published:** 2025-07-17

**Authors:** Bizhi Tu, Run Fang, Peizhi Lu, Mingxiang Liu, Shuo Yang, Dingtao Hu, Renzhi Ruan, Rende Ning

**Affiliations:** a Department of Orthopedics, The Third Affiliated Hospital of Anhui Medical University (The First People’s Hospital of Hefei), Hefei, Anhui, China; b Department of Oncology, The First Affiliated Hospital of Anhui Medical University, Hefei, Anhui, China

**Keywords:** aging, biomarkers, machine learning, osteoarthritis, single-cell RNA sequencing

## Abstract

**Background:**

Osteoarthritis (OA) pathogenesis involves age-related immune dysregulation, yet non-invasive diagnostic tools and mechanistic insights remain limited.

**Methods:**

We integrated transcriptomic profiling of four OA-affected joint tissues with machine learning to identify potential peripheral blood biomarkers. Weighted gene co-expression network analysis was employed to explore gene modules and pathways associated with OA. Single-cell RNA sequencing was performed on 217 983 joint cells to delineate B cell differentiation trajectories. Flow cytometry was used to validate age-associated B cell imbalances in peripheral blood.

**Results:**

We identified five peripheral blood biomarkers: MAPK1, MAP3K8, ING1, LDLR, and NUP153 in distinguishing OA patients from controls (the area under the curve [AUC] = 0.966). Importantly, these markers exhibited age-specific expression profiles; ING1, NUP153, and MAP3K8 were elevated, while MAPK1 was reduced in elderly compared to younger OA patients. A refined predictive model based on these age-specific markers demonstrated superior performance specifically for elderly Knee OA (KOA, AUC: 0.8 vs. 0.7 for younger KOA). These biomarkers correlated with immune cell infiltration and inflammatory cytokines. In osteoarthritic joint tissues, B cells predominantly originated from subchondral bone and synovium. Single-cell analysis identified age-specific B cell differentiation patterns, with elderly KOA patients enriched in an activated B cell cluster (C1). Furthermore, B cells from elderly KOA patients showed altered energy metabolism and increased proportions in peripheral blood, and functionally promoted chondrocyte damage.

**Conclusion:**

Our findings establish a novel blood-based diagnostic framework for OA and uncover aging-driven B cell remodeling as a key contributor to elderly OA pathogenesis. These findings offer non-invasive diagnostics and immunomodulatory targets for age-specific OA therapy.

**HIGHLIGHTS**

- Identifies a novel 5-gene blood biomarker panel (MAPK1, MAP3K8, ING1, LDLR, and NUP153) for non-invasive OA diagnosis.
- Reveals age-specific biomarker signatures enabling elderly focused diagnostics (AUC = 0.8), with ING1/NUP153/MAP3K8 upregulated and MAPK1 downregulated in elderly OA.
- Uncovers aging-driven B cell remodeling in OA joints: elderly patients show enriched activated B cells (Cluster C1) with altered mitochondrial metabolism.
- Demonstrates B cells from elderly OA promote chondrocyte damage, implicating age-skewed B cell responses as key therapeutic targets.

## Background

Osteoarthritis (OA) is a degenerative disease characterized by articular degeneration^[1,2]^ that becomes increasingly prevalent with prolonged life expectancy. This chronic progressive condition imposes substantial psychological and disability burdens on over 240 million patients worldwide^[3]^. Elderly individuals, particularly those over 65 years old, experience pain and disability primarily due to OA^[4]^. The global burden of disability associated with OA accounts for 2.4% of all years lived with disability, with a 75% increase observed between 1990 and 2013^[5]^. Notably, OA-related symptoms such as depressed mood, fatigue, and poor-quality sleep significantly impair the quality of life^[6]^. Moreover, symptom severity tends to worsen over time^[7]^, and the absence of effective disease-modifying drugs often necessitates knee replacement surgery^[8]^. Consequently, there is an urgent need to explore potential mechanisms and therapeutic targets to improve the quality of life for OA patients.

The characteristic of pathologic changes of OA includes cartilage destruction, meniscus injury, synovitis, subchondral bone alterations, and other extra-joint factors^[9]^. Preventing articular cartilage loss and mitigating low-grade synovitis are critical to delaying joint degeneration^[10–12]^. Meniscus defects can initiate joint inflammation, while subchondral sclerosis often manifests in advanced OA stages^[13,14]^. While previous studies have primarily focused on biomarkers specific to individual joint components (cartilage, synovium, meniscus, or subchondral bone)^[15–17]^, few reports have investigated biomarkers encompassing these four components collectively. Therefore, a comprehensive understanding of OA pathology should consider the degeneration of all four articular components^[18]^. Furthermore, therapies targeting single joint components may not yield optimal efficacy^[10]^, underscoring the importance of investigating alterations across multiple components to elucidate potential mechanisms underlying joint destruction in OA^[19]^. To address this gap, our study employs advanced machine learning techniques to identify blood-based biomarkers across all four joint components, ensuring transparency in artificial intelligence (AI) use in accordance with the TITAN Guidelines 2025^[20]^.

Recent research has shifted from the traditional “mechanical wear” model to one focused on immune-inflammatory regulation^[21]^. Increasing evidence indicates that systemic^[22]^ and local immune-inflammatory disruptions, particularly chronic low-grade synovitis, are central to driving joint degeneration, pain, and dysfunction^[23,24]^. Persistent inflammatory microenvironment accelerates joint structural damage by inducing chondrocyte death and disrupting the balance between osteogenesis and osteoclastogenesis, which promotes subchondral bone sclerosis and osteophyte formation^[25]^. Moreover, the chondrogenic differentiation of bone marrow mesenchymal stem cells is tightly regulated by dynamic cellular crosstalk within the articular microenvironment^[26]^. B lymphocytes are key regulators of joint homeostasis, with their dysregulated infiltration and phenotypic shifts mechanistically linked to joint inflammation and degeneration^[27–29]^. The transition of B cells to long-lived antibody-secreting phenotypes is a critical driver of the persistent chronic low-grade inflammatory response within the joint^[30,31]^. Despite accumulating evidence suggesting that aberrant B cell activation (characterized by a shift toward an antibody-secreting phenotype or immune-regulatory subsets such as Bregs^[32,33]^) contributes to the pathogenesis of OA, age-related changes in B cell populations also play a role in intra-articular inflammation^[34]^. However, the relationship between age-related B cells and OA remains to be further elucidated.

Despite some reports identifying potential biomarkers for OA, reliable diagnostic biomarkers identified from peripheral blood samples remain scarce^[17,18]^. This study identified differentially expressed OA-related hub genes in the four joint components (cartilage, synovium, meniscus, and subchondral bone) and explored the probability of these genes as blood markers for predicting OA. Our findings demonstrate that these hub genes have potential as robust blood-based biomarkers for diagnosing OA. Additionally, the expression of these hub genes may be associated with immune dysregulation in the peripheral blood of OA patients.

## Materials and methods

### Bioinformatic analysis

The methods of bioinformatic analysis were presented in the Supplementary material. Machine learning algorithms, including random forest, Support Vector Machine-Recursive Feature Elimination (SVM-RFE), and multivariate regression, were employed to identify potential blood biomarkers for OA. These AI-based methods were used to analyze transcriptomic data, as detailed in the Supplementary material, in compliance with the TITAN Guidelines 2025 for transparency in AI reporting^[20]^.

### Blood sampling and flow cytometry

Blood samples were obtained from end-stage knee OA patients undergoing total knee arthroplasty (TKA). Informed consent was obtained from all participants prior to their enrollment in the study. Peripheral blood mononuclear cells (PBMCs) were isolated using standard Ficoll (GE Healthcare) centrifugation techniques. The following antibodies were utilized: APC/cy7-conjugated anti-human CD19 (eBioscience, A15429), APC-conjugated anti-human CD27 (BioLegend, 302809), PE-conjugated anti-human IgM (eBioscience, 12-9998-42), AF700-conjugated anti-human CD24 (eBioscience, 56-0049-42), and PE/cy7-conjugated anti-human CD38 (eBioscience, 25-0389-42). These antibodies (0.5 μL for each antibody) were added to 100 μL of PBMCs and incubated for 30 min at 4°C in the dark. Subsequently, the cells were washed twice with phosphate-buffered saline (PBS). Instrument settings and cell gating procedures were based on established protocols to isolate the cells accordingly, followed by the analysis of lymphocyte surface molecule expression. Results are presented as the percentage of cells expressing specific markers.

### Quantitative PCR

Quantitative PCR (qPCR) was performed to measure the expression levels of the screened genes in blood samples from donors with or without OA. Primers specific to screened gene and the housekeeping gene GAPDH were designed using Primer-BLAST (NCBI) and synthesized by Integrated DNA Technologies. Each qPCR reaction was conducted in triplicate using the PowerUp™ SYBR Green Master Mix (Applied Biosystems) in a final volume of 20 μL, containing 10 ng of cDNA, 0.5 μM of each primer, and nuclease-free water. The qPCR protocol included an initial denaturation step at 95°C for 10 min, followed by 40 cycles of 95°C for 15 s and 60°C for 1 minu. The reactions were carried out on a QuantStudio™ 7 Flex Real-Time PCR System (Applied Biosystems). The relative expression of the target genes (fold change) was determined by 2^−ΔΔCT^, where ΔCT = CT(target) − CT(GAPDH), and ΔΔCT = meanΔCT(Elderly group)  − ΔCT(Younger group).

### Chondrocyte isolation and culture

Cartilage tissues were harvested from patients undergoing TKA. The excised cartilage was immersed in PBS, minced into small pieces, and evenly distributed along the sidewall of a cell culture flask. The flask was supplemented with DMEM medium (Gibco, USA; Cat# 62247) containing 20% fetal bovine serum, 100 µg/mL streptomycin, and 100 U/mL penicillin. The flask was incubated upright for 8 h and then placed horizontally to ensure complete submersion of the tissue in the medium. After 1 week, chondrocytes adhering to the flask bottom were collected and passaged (P3–P8) for subsequent experiments.

### Isolation of Naïve B cells

Peripheral blood was collected from healthy volunteers. PBMCs were isolated by density gradient centrifugation using human lymphocyte separation medium (Dakewe, China; Cat# 7111011). Naïve B cells and total B cells were then isolated from PBMCs using the Human Naïve B Cell Isolation Kit (Stemcell, Canada; Cat# 17254) and the Human B Cell Isolation Kit (Stemcell, Canada; Cat# 17954), respectively, following the manufacturers’ protocols.

### Co-culture and protein analysis

Approximately 10 000 chondrocytes were co-cultured with 10 000 peripheral blood-derived B cells from healthy controls, younger KOA patients, or elderly KOA patients in six-well plates for 2 days. The synthetic and catabolic activities of chondrocytes were assessed by Western blotting using antibodies against MMP13 (Abcam, UK; Cat# b39012) and COL2 (Abcam, UK; Cat# ab307674).

### Statistical analysis

The statistical analysis was done using SPSS software (version 23.0), while raw data processing was conducted in R software (version 4.2.1). For pairwise comparisons, normality and homogeneity of variance are first assessed. If both assumptions are satisfied, a *t*-test is used; otherwise, the Wilcoxon test is applied to explore the difference between OA and CT groups. A two-tailed *P*-value less than 0.05 indicates a statistically significant difference.

## Results

### Identification of osteoarthritic-related genes in OA

**Figure 1.**
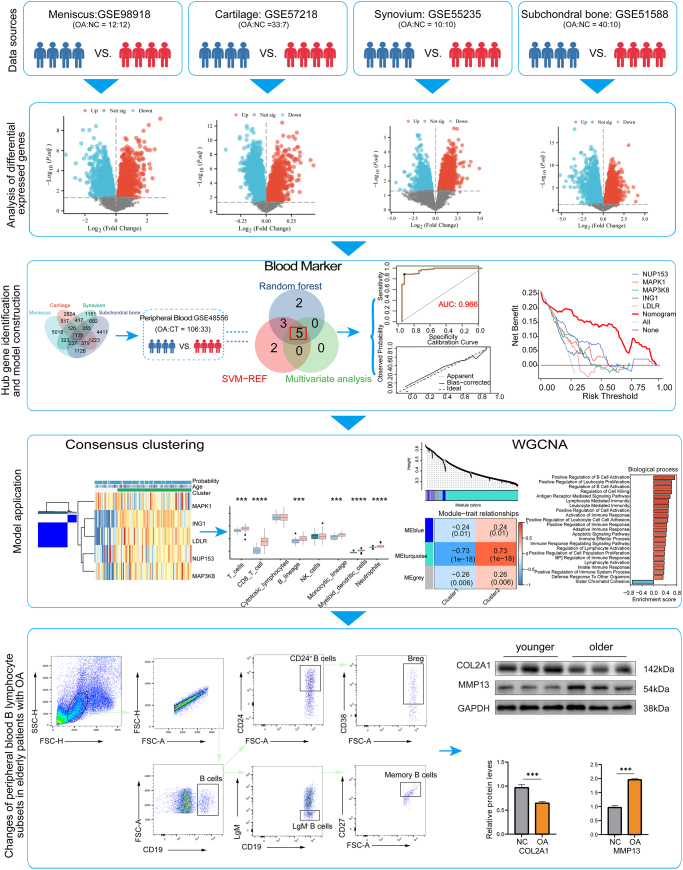
Flow diagram for comprehensive analysis of blood markers associated with knee articular degeneration in osteoarthritis.

The flow diagram of analysis procession is presented in Figure 1. All AI outputs were reviewed and validated by the corresponding author and senior authors. No generative AI tools were used in the writing or editing of the manuscript, and figure creation. All authors take full responsibility for the integrity of the results.

**Figure 2.**
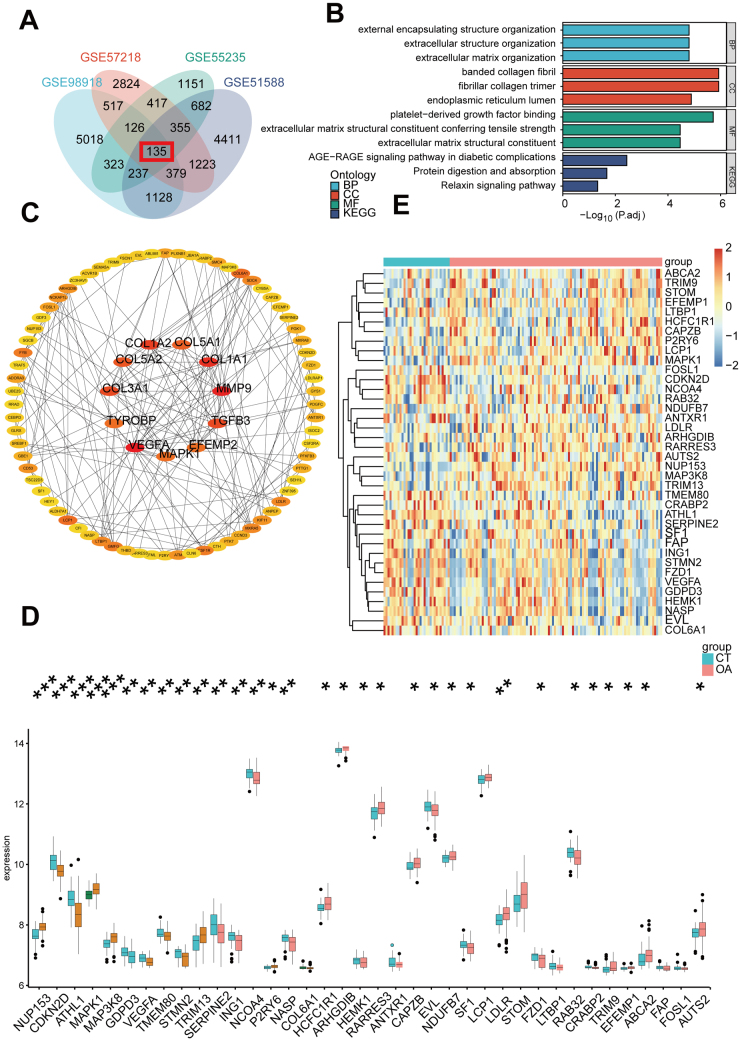
Identification of common differentially expressed genes (co-DEGs) associated with extensive destruction in OA joint components. (A) Identification of co-DEGs in four joint components in OA. GO/KEGG (B) and protein–protein interaction (PPI) network (C) analysis of identified co-DEGs. (D) Identification of co-DEGs both in four joint components and peripheral blood. (E) Heat map of expression heterogeneity of co-DEGs between OA and healthy control. *P < 0.05, ** *P* < 0.01, ***P < 0.001.

To systematically identify osteoarthritic-related genes in OA, we performed differential expression analysis across four joint components (meniscus, synovium, subchondral bone, and cartilage) using the *limma* package in R (Supplemental Digital Content, Fig. S1, available at: http://links.lww.com/JS9/E670). The detailed information for patients obtained from the GEO database was shown in Supplemental Digital Content Table S1. We obtained a dataset of 10 513 genes encoding secreted proteins from the GeneCards database (https://www.genecards.org/). Venn analysis revealed 135 overlapping differentially expressed genes (DEGs) encoding secreted proteins consistently dysregulated in all four tissues (Fig. 2A), designated as osteoarthritic-related genes. Then, these osteoarthritic-related genes were used to perform the Gene Ontology/ Kyoto Encyclopedia of Genes and Genomes(GO/KEGG) analysis and conduct the Protein-Protein Interaction (PPI) network. As shown in Figure 2B, the biological process of osteoarthritic-related genes mainly enriched in “extracellular matrix organization”, “external encapsulating structure organization”, and “extracellular structure organization”, among the cellular-component categories, the osteoarthritic-related genes were significantly enriched in “collagen fibril” and “fibrillar collagen trimer”; the most obvious enrichment of osteoarthritic related genes in molecular function were “platelet-derived growth factor binding”; and the “AGE-RAGE signaling pathway in diabetic complications” was most obvious in KEGG. More details were presented in Supplemental Digital Content Table S2. The constructed PPI network is presented in Figure 2C. The external peripheral blood-related dataset GSE48556 (Fig. 2D) was used to explore whether these overlapping DEGs were expressed differentially in the peripheral blood of OA patients. The expression level of 38 osteoarthritic-related genes was found statistically different in the peripheral blood of OA. The heat map displays the expression heterogeneity of these potential blood markers between CT and OA samples in GSE48556 (Fig. 2E).

### Peripheral blood biomarkers for OA diagnosis

**Figure 3.**
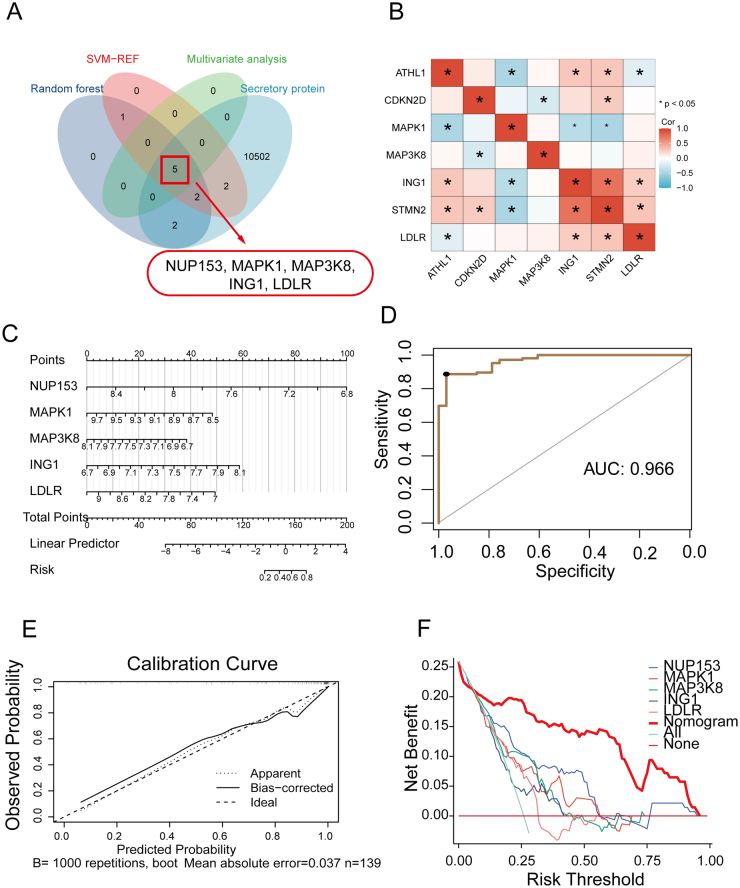
Identification of diagnosis-related blood marker in OA. (A) Five hub genes were identified by random forest, SVM-RFE, and multivariate analysis. (B) Expression level of five hub genes in peripheral in OA. (C) Nomogram of five hub genes in the diagnosis of OA patients. (D) Receiver operating characteristic (ROC) curve of predicted risk scores in OA diagnosis. (E) Calibration curve for the diagnostic model. (F) Model evaluation curves: a diagnostic model containing multiple nominated hub genes makes patients more profitable than a single gene.

Transcriptomics-identified hub DEGs demonstrate biomarker potential for OA^[17,33,35]^. Given the impracticality of invasive joint sampling for routine OA diagnosis, we expected that peripheral blood-based detection of these OA-related genes could enhance clinical utility. Thus, peripheral blood transcriptomes from 106 OA patients and 33 healthy controls were analyzed by random forest (Supplemental Digital Content, Fig. S2A, available at: http://links.lww.com/JS9/E670), SVM-RFE (Supplemental Digital Content, Fig. S2B, available at: http://links.lww.com/JS9/E670), and multivariate regression (Supplemental Digital Content, Fig. S2C, available at: http://links.lww.com/JS9/E670), we identified the five most significant osteoarthritic-related genes (potential blood markers) in the peripheral blood of OA patients: Mitogen-Activated Protein Kinase 1 (MAPK1), Mitogen-Activated Protein Kinase Kinase Kinase 8 (MAP3K8), Inhibitor of Growth Family Member 1 (ING1), Low-Density Lipoprotein Receptor (LDLR), and Nucleoporin 153 (NUP153) (Fig. 3A). The expression of MAPK1, MAP3K8, LDLR, and NUP153 was up-regulation, and the expression of ING1 was down-regulation in peripheral blood of patients with OA (Fig. 3B, *P* < 0.05 for all). We developed a diagnostic model for OA based on the expression of blood markers (Fig. 3C). The nomogram shows that the total scores determined the possibility of OA. The model’s AUC presents a more excellent diagnostic efficacy (Fig. 3D, AUC = 0.966) than one marker (Supplemental Digital Content, Fig. S2D, available at: http://links.lww.com/JS9/E670). Linear calibration curves show that the prediction model was appropriate (Fig. 3E). The ROC curve’s credibility was validated through bootstrap resampling with replacement from the OA samples. (Supplemental Digital Content, Fig. S2E, available at: http://links.lww.com/JS9/E670, n = 100 bootstraps). Supplemental Digital Content Figures S2F, S2G, and S2H, available at: http://links.lww.com/JS9/E670, depict the distribution ranges of AUC, specificity, and sensitivity, respectively. Compared with a single hub gene, the model brings better benefits for OA patients (Fig. 3F).

### Consensus clustering reveals age-specific blood biomarker signatures in OA

**Figure 4.**
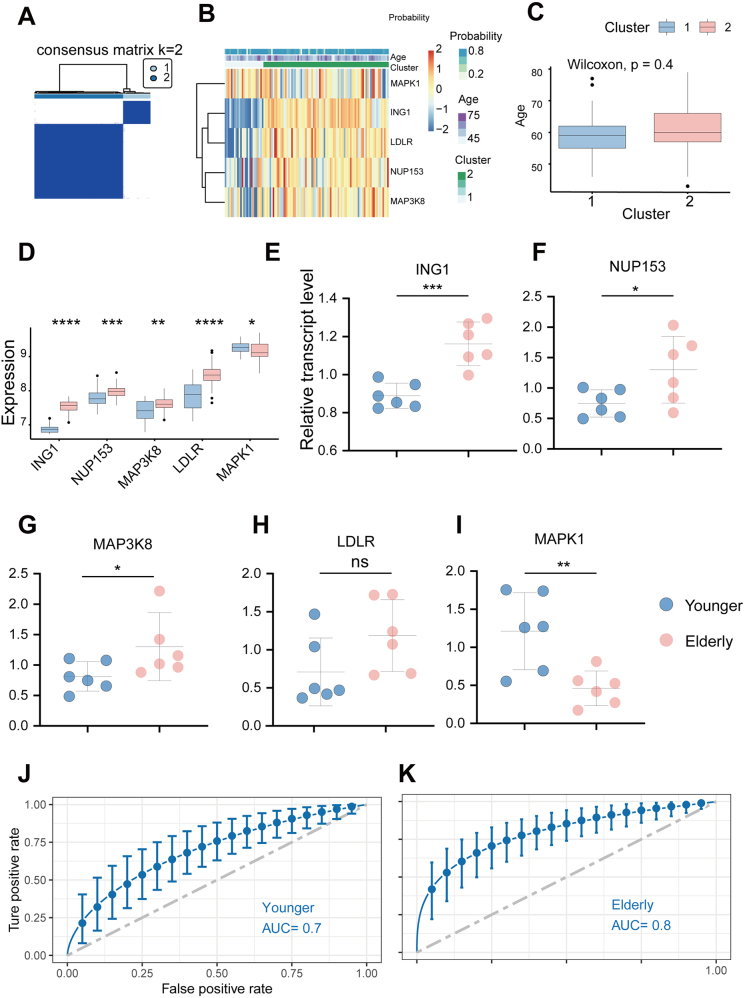
Age-stratified molecular profiling and diagnostic performance of blood biomarkers in OA. (A) Consensus matrix plots depicting consensus values on a white-to-blue color scale ordered by consensus clustering when two clusters were selected. (B) The heterogeneity of gene expression is related to age. (C) Age difference between two clusters. (D) Expression differences of five hub genes between two clusters. Differential expression of ING1 (E), NUP153 (F), MAP3K8 (G), LDLR (H), and MAPK1 (I) in peripheral blood of elderly versus younger OA patients. ROC analysis of peripheral blood biomarkers (ING1, NUP153, MAP3K8, and MAPK1) for discriminating younger (J) versus elderly (k) KOA onset. *P < 0.05, **P < 0.01, ***P < 0.001, ****P < 0.0001.

We performed consensus cluster analysis on OA patients based on the expression of five blood markers, resulting in their classification into two distinct clusters (Fig. 4A). The connection between the expression of blood markers, age, and immune characteristics was explored in R software with the “ggpubr” package. As present in Figure 4B, the level of blood markers, age, and OA possibility between two clusters were significantly heterogeneous. Cluster 2 exhibited a higher proportion of elderly OA patients compared to cluster 1 (Fig. 4C). Four of five markers (MAP3K8, ING1, LDLR, and NUP153) were found significant higher in cluster 2 (elderly population) than in cluster 1 (Fig. 4D). To validate the expression levels of selected blood markers in elderly OA patient samples, qPCR analysis was performed. The primer sequences used are detailed in Supplemental Digital Content Table S3. Our findings align closely with previous results, indicating that in elderly OA patients, ING1, NUP153, and MAP3K8 expression levels were elevated (Fig. 4E, 4F, 4G), while MAPK1 expression is reduced (Fig. 4I) when compared to younger OA patients. The only exception was LDLR, where qPCR did not reveal any significant changes (Fig. 4H), deviating from the bioinformatic predictions. To validate the clinical efficacy of the identified blood biomarkers, peripheral blood samples were collected from three cohorts: healthy controls (*n* = 48), younger KOA patients (*n* = 48), and elderly KOA patients (*n* = 48) (Supplemental Digital Content Table S4). Since LDLR gene expression showed no significant differences between young and elderly KOA patients (Fig. 4H, *P *> 0.05), we constructed a predictive model based on the remaining four biomarkers. Consistent with our hypothesis, the selected biomarkers demonstrated superior predictive performance specifically for elderly KOA patients (AUC: 0.8 vs. 0.7, Fig. 4J, 4K).

### Immune dysregulation linked to OA-related blood markers

**Figure 5.**
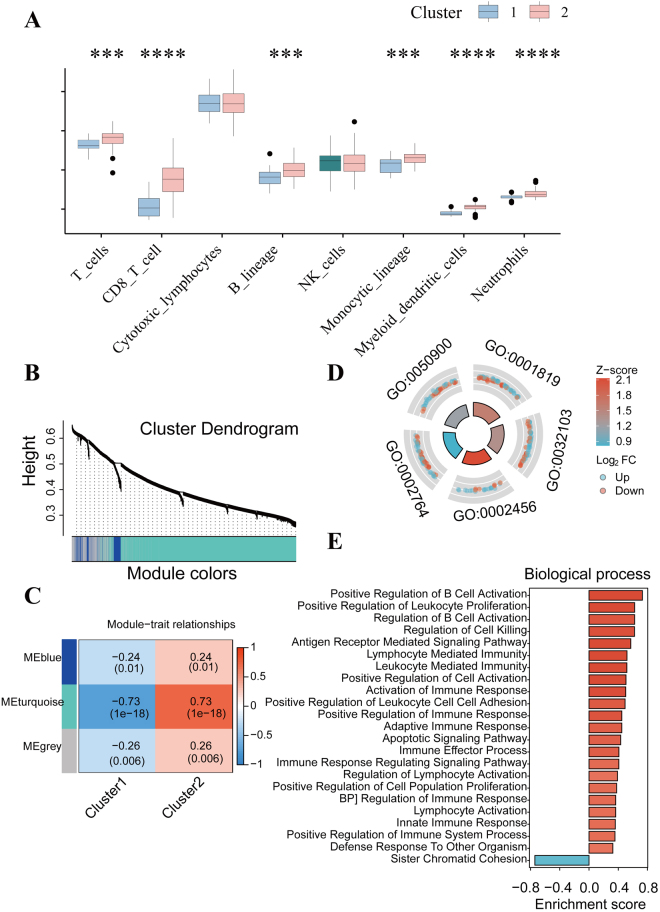
The results of unsupervised consensus clustering based on identified hub genes. (A) The difference of immune cells’ abundance between two clusters. (B) Clustering dendrogram of genes based on topological overlapping. (C) Heatmap of the correlation between module eigengenes and two clusters. Biological process from GO (C) and GSEA (E) for two clusters that most likely enriched in the turquoise model. ***P < 0.001, ****P < 0.0001.

The pathogenesis of OA is related to immune disorders and low-grade inflammation^[36]^. Thus, we comprehensively analyzed the correlation between immune cell infiltrations, inflammatory factors, and the expression of five blood markers in OA’s peripheral blood. Through the CIBERSORT and MCP-counter analysis (Supplemental Digital Content, Fig. S3A, S3B, S3C, available at: http://links.lww.com/JS9/E670), we found that ING1 was significantly correlated with 8 of 16 immune cell infiltrations, the abundance of 6 of 8 immune cell populations, and the level of 24 of 28 inflammatory cytokines. For NUP153, we observed that NUP153 was negatively correlated with 2 of 16 immune cell infiltrations, the abundance of 4 of 8 immune/stromal cell populations, and the level of 15 of 28 inflammatory cytokines. The expression level of MAP3K8 was positively associated with T regulatory cell infiltration, the abundance of 2 of 8 immune cell populations, and the level of 5 of 28 inflammatory cytokines. For LDLR, we found that LDLR expression was associated with 2 of 16 immune cell infiltrations, the abundance of 3 of 8 immune cell populations, and the level of 17 of 28 inflammatory cytokines. For MAPK1, we found that MAPK1 expression was associated with 11 of 16 immune cell infiltrations, the abundance of 6 of 8 immune cell populations, and the level of 18 of 28 inflammatory cytokines. Moreover, a higher abundance of T_cells, CD8_T_cells, B_lineage, Monocytic_lineage, Myeloid_dendritic_cells, and Neutrophils was found in the peripheral blood of cluster 2 (Fig. 5A) and the expression level of 22 of 28 inflammatory cytokines was found significant differentially expressed between two clusters (Supplemental Digital Content, Fig. S3D, available at: http://links.lww.com/JS9/E670).

### Weighted gene co-expression network analysis identifies immune-centric co-expression modules

Quality checks were conducted on 106 peripheral blood samples obtained from patients with OA, and no sample was excluded during the process (Supplemental Digital Content, Fig. S4A, available at: http://links.lww.com/JS9/E670). Co-expression modules were established using dynamic tree-cut analysis. The scale-free network was constructed after setting the optimal soft threshold (Supplemental Digital Content, Fig. S4B, available at: http://links.lww.com/JS9/E670). Using weighted gene co-expression network analysis (WGCNA), all co-expression modules with statistical significance were identified based on optimal dynamic tree cut and hierarchical clustering (Fig. 5B). The relationship between the two clusters and clinical phenotypes was explored. Contrary to cluster 1, we found that cluster 2 was positively related to the “turquoise” module (Fig. 5C). Then, the model genes that are most relevant to cluster 1 or 2 were applied to conduct an enrichment analysis of the biological process. As shown in Figure 5D and Supplemental Digital Content Table S5, the most relevant model “turquoise” was enriched in immune-related pathways, such as T cell-mediated immunity (GO:0002456), immune response-regulating signaling pathway (GO:0002764), and leukocyte migration (GO:0050900). Moreover, the Gene Set Enrichment Analysis (GSEA) was run to further explore the different biological process of “turquoise” module genes. Significantly, older OA patients in cluster 2 exhibited distinct B lymphocyte abnormalities in peripheral blood, including “Positive Regulation of B Cell Activation” and “Regulation of B Cell Activation,” in contrast to patients in cluster 1 (younger) (Fig. 5E).

### B cell subpopulations and their evolutionary trajectories in OA

**Figure 6.**
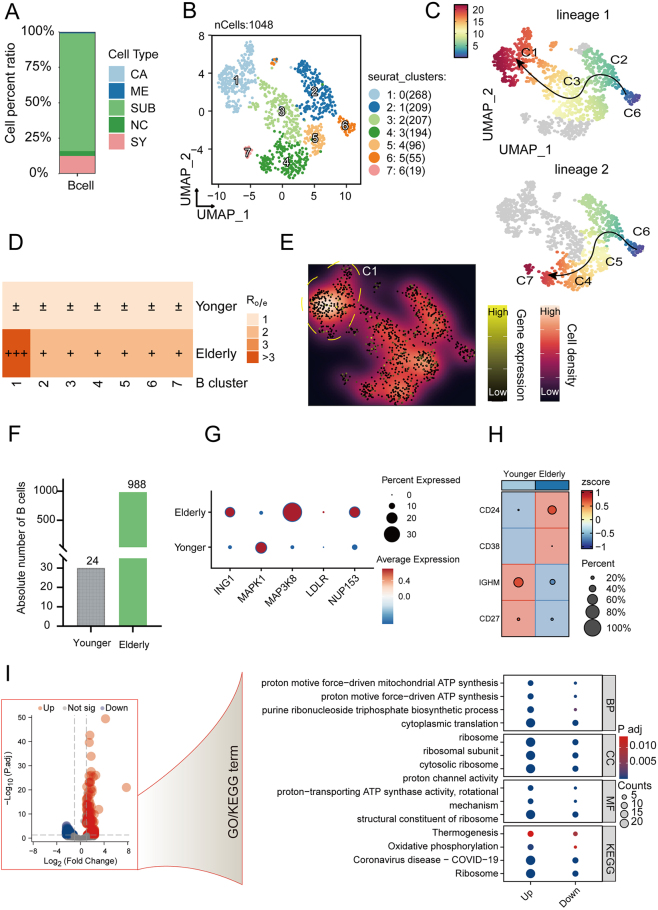
Integrative profiling reveals age-dependent B-cell remodeling in osteoarthritis joints. (A) Proportional distribution of B cells across the four joint components in osteoarthritis. NC: normal control, CA: osteoarthritic cartilage, ME: osteoarthritic meniscus, SUB: osteoarthritic subchondral bone, SY: osteoarthritic synovium. (B) UMAP visualization of B cells in joint of patients with OA. (C) Pseudotime trajectory analysis of B cell subpopulations performed with the slingshot algorithm, shown on a UMAP plot, where different colors represent distinct B cell subgroups. (D) Propensity of B cells in young and elderly KOA tissues. Ro/e > 3 indicates significant enrichment of the cell subpopulation within that tissue. (E) Overall expression levels of hub osteoarthritic-related genes across B cell subpopulations. The region demarcated by the white dashed box corresponds to the C1 subpopulation, which exhibits both increased cellular abundance and significantly elevated expression levels of hub osteoarthritic-associated genes. (F) Frequency of B cells in patient-derived lymphocytes comparing younger and elderly individuals with OA. (G) Differential expression of hub osteoarthritic-related genes in younger versus elderly patients with OA. (H) Differential surface expression of B-cell differentiation and activation markers (CD24, CD38, IgM, and CD27) in patient-derived B cells from younger versus older individuals with OA. (I) Volcano plot depicting transcriptomic profiles and functional enrichment analysis of differentially expressed genes (DEGs) in B cells derived from younger versus elderly OA patients.

To investigate the differentiation trajectories of B cells in OA and their association with OA-related genes, we analyzed single-cell data from four joint components in OA (Supplemental Digital Content Table S6). A total of 217 983 cells were included for subsequent analysis. Through dimensionality reduction and clustering, we identified 25 distinct cellular populations (Supplemental Digital Content, Fig. S5A, available at: http://links.lww.com/JS9/E670). Among them, 1048 B cells (cluster 17) were identified based on lineage-specific gene expression and expression of B cell markers CD19 and MS4A1 (Supplemental Digital Content, Fig. S5B, available at: http://links.lww.com/JS9/E670). We observed that osteoarthritic B cells predominantly originated from subchondral bone and synovial tissues (Fig. 6A), We then performed dimensionality reduction and clustering analysis on 1048 identified B cells, revealing seven distinct B cell clusters (Fig. 6B). Using the Slingshot algorithm for trajectory inference, we identified two separate differentiation trajectories for B cells. Cluster C6 marked the activation and differentiation starting point, while clusters C1 and C7 represented the endpoints of the two distinct differentiation pathways (Fig. 6C). We conducted GO enrichment analysis on the highly expressed genes (Supplementary material for DEGs of B clusters) within each B cell cluster (DEGs in the B cell clusters are provided in Supplemental Digital Content Figure S6C, available at: http://links.lww.com/JS9/E670). Cluster C7 primarily exhibited RNA-level changes, enriched in mRNA processing and RNA splicing pathways. Cluster C1 was enriched in B cell activation, lymphocyte and mononuclear cell proliferation, and leukocyte proliferation. Ratio of observed to expected (Ro/e) analysis revealed that the C1 subset, as the terminal differentiation state, was significantly enriched in elderly KOA patients (Fig. 6D), indicating an altered differentiation capacity of B cells in the joints of elderly KOA patients. Furthermore, OA-related genes were relatively more highly expressed in the C1 subset (Fig. 6E). To further explore the differences in B cell differentiation and activation, as well as the expression of OA-related genes in young versus elderly KOA patients, we first determined that the majority of B cells in KOA patients were derived from the elderly group (Fig. 6F). Based on the expression levels of OA-associated genes, we found that ING1, MAP3K8, and NUP153 were significantly upregulated in elderly KOA, while MAPK1 was downregulated (Fig. 6G). We also observed distinct expression levels of B cell activation and differentiation markers between young and elderly KOA patients (Fig. 6H). To explore the functional differences in B cells between young and elderly KOA patients, we conducted GO/KEGG enrichment analysis of the differential genes in both groups. The results (Supplemental Digital Content, Table S7) revealed significant alterations in energy metabolism such as “proton motive force−driven mitochondrial ATP synthesis” and “Oxidative phosphorylation” in B cells from elderly KOA patients (Fig. 6I).

### Flow cytometry confirms age-associated B cell shifts in OA

**Figure 7.**
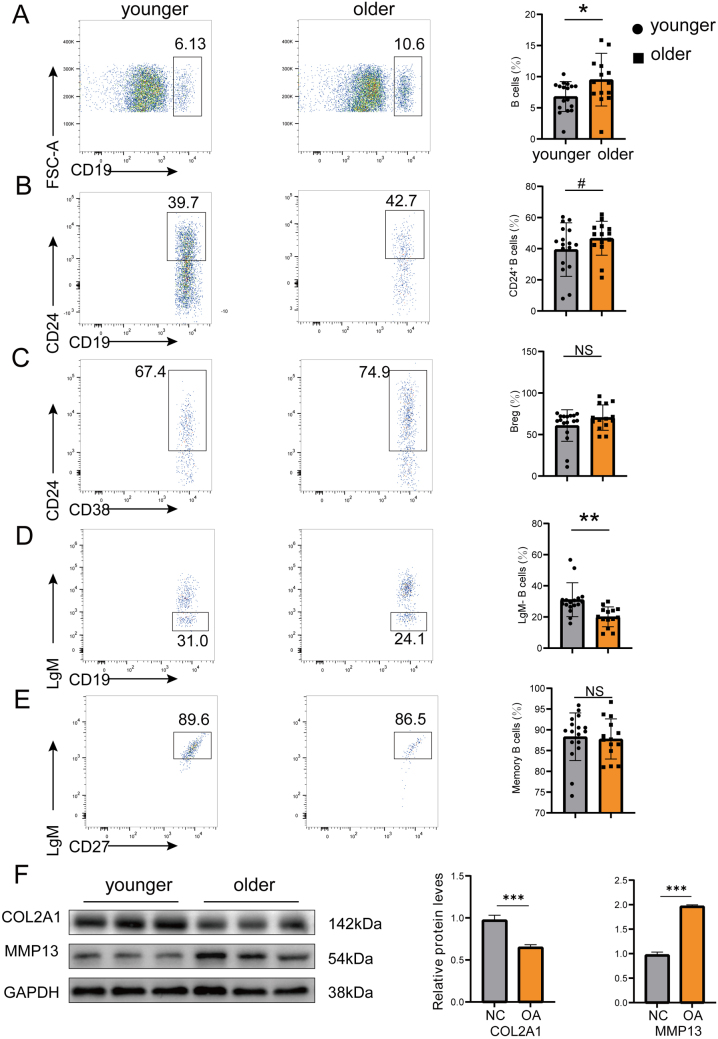
Age-related differences in peripheral blood B cell subsets and their functional impact on chondrocytes in osteoarthritis. The frequency of total B cells (A), CD24^+^ B cell (B), LgM^−^ B cell (C), regular B cell (Breg, D), and memory B cell (E) in peripheral blood of younger and older patients with OA. (F) Catabolism (MMP13) and anabolism (COL2A1) changes in chondrocytes co-cultured with peripheral blood B cells from younger and elderly OA patients. *P < 0.05, **P < 0.01, ***P < 0.001, #*P* = 0.057. NS, not significant.

Existing research suggests that advancing age may diminish the differentiation and antibody production capacity of B lymphocytes^[37,38]^. To further investigate whether changes in B cell subset distribution in peripheral blood of older patients with OA are more pronounced, we conducted B cell subset analysis of PBMCs from 17 older and 14 younger late-stage KOA patients with flow cytometry. Gating strategies of the B cell subset are shown in Supplemental Digital Content Fig. S5, available at: http://links.lww.com/JS9/E670. As shown in Figure 7A, we observed a significant increase in the proportion of total B cells in the peripheral blood of older OA patients. Regarding B cell maturation, elderly OA patients show an increasing trend in peripheral blood CD24^+^ B cells (Fig. 7B) and a decreasing trend in IgM^−^ B cells (Fig. 7C). As for B cell differentiation, no significant differences were observed in the differentiation of memory B cells (Fig. 7D) and regulatory B cells (Bregs, Fig. 7E) between younger and elderly OA patients in peripheral blood. To determine whether senescent B cells contribute to articular cartilage damage, we co-cultured peripheral blood B cells from younger (<65 years old) and elderly (>65 years old) healthy donors with primary chondrocytes. Our findings demonstrate that B cells derived from elderly patients significantly promoted the expression of COL2A1 and suppressed the expression of MMP13 in chondrocytes, compared to those from young patients (Fig. 7F).

## Discussion

Previous studies have revealed potential diagnostic genes expressed differentially among two or more joint components^[19]^. However, the diagnostic potential of hub genes that are widely expressed in four joint components (meniscus, cartilage, synovium, and subchondral bone) has been uncovered. In this study, we identified five blood markers that were widely differentially expressed in four joint components, and these blood markers can serve as potential diagnostic markers for OA. Additionally, we conducted a comprehensive analysis and found that the blood markers widely affect the immune landscape of peripheral blood in OA patients.

Extracellular matrix degradation of articular cartilage is a well-recognized factor in the development and progression of OA^[39,40]^. Advanced glycation end product (AGE) is an irreversible and stable macromolecular complex that participates in OA progression, including accelerating degradation of extracellular matrix and promoting apoptosis of chondrocytes^[41,42]^. In the current study, the 135 overlapping DEGs widely expressed in four OA joint components were mainly enriched in the GO extracellular structure-related pathway, such as extracellular matrix and collagen. The KEGG analysis shows that the AGE-RAGE signaling-related pathway was found significantly enriched. Nutraceutical supplements derived from collagen have been reported to have potential efficacy in inflammation-induced joint swelling^[43]^. Pretreatment with RAGE antagonist in rabbit chondrocytes can greatly reverse the TNF-α and MMP-13 expressions induced by AGE treatment^[44]^. The AGE binds to its receptor RAGE and then enhances oxidative stress, activates the NF-κB signaling pathway, and stimulates cytokine production and growth factors, thereby causing damage to intra-articular cells and tissues^[45]^. As for meniscus, AGE-RAGE activation activates mTOR, simultaneously promoting the accumulation of ATF4, thereby enhancing the ATF4-mTOR positive feedback loop, which in turn strengthens the osteogenic potential of meniscal cells^[46]^. However, decreasing the level of AGE and their receptors may not improve the clinical endpoints of OA^[47]^. These inconsistent results may stem from differential regulatory roles of AGE in various components within the joint. Additionally, the articular cartilage has no nerves or blood vessels, and its stable homeostasis mainly depends on the local microenvironment^[48,49]^. Hence, improving the local microenvironments, such as reversing the extracellular matrix remodeling and collagen degeneration, may be a promising therapeutic method in OA.

Timely identification and intervention of OA are imperative in preventing the loss of cartilage and degeneration of joints.^[10,11]^. However, the initial manifestations of OA lack specificity, and patients in early stages often miss opportunities for timely clinical intervention. Previous studies reveal some novel potential osteoarthritic diagnostic signatures. However, the constructed diagnostic model has been limited to hub genes identified from a single joint component^[33,35,50]^. However, as OA is an age-related degenerative joint disease with significant intra-articular heterogeneity^[51,52]^, reliable blood-based biomarkers specific for predicting elderly-onset OA had not been established prior to this study. Notably, our study identified four peripheral blood biomarkers that demonstrate superior predictive performance for elderly OA onset compared to younger OA cases (AUC: 0.8 vs. 0.7). Therefore, clinical application of routine screening using intra-articular tissue may not be practical. In the current study, we included the five hub genes that were differentially expressed in four joint components and peripheral blood in OA. Our constructed model may reveal a real possibility that screens for the early onset of OA only through blood testing.

Previous studies reveal that MAPK1 aggravates the chondrocyte injury induced by inflammatory factors IL-6^[53]^, and MAP3K8 promotes the OA progression^[54]^. In the current study, compared to normal tissues, we found higher expression of MAPK1 and MAP3K8 in the peripheral blood of OA patients. Consistent with previous reports, our results indicated the adverse role of MAPK1 and MAP3K8 in OA (OR > 1). Micro-articular damage, leading to exposure of the extracellular matrix and subsequent activation of innate immunity, has been identified as a primary contributor to the progression of OA^[55]^. ING1 has a potential role in maintaining cell–cell junction integrity^[56]^. Thus, ING1 may have a protective function in the integrity of the joint microstructure (OR < 1). The nuclear pores, built with the evolutionarily conserved nucleoporin complex (NUP), assist the nucleocytoplasmic transport of biomolecules^[57]^. NUP153, as a member of the NUP protein family, regulates the developmental differentiation of cells and proliferative invasion of tumors^[58,59]^. In the current study, the NUP153 expression was significantly up-regulated in OA patients’ peripheral blood and was higher in the elderly group. Greater attention/focus should be directed to NUP153 expression in older OA patients. The dysregulation of lipid metabolism in patients with OA has garnered increasing attention. LDLR is one of the key molecules for maintaining homeostasis of lipid metabolism^[60]^. Lipid metabolism disorder leads to persisting low-grade systemic inflammation that increases inflammation in OA^[36]^. In this study, higher expression of LDLR in the elderly population of OA may indicate a more severe lipid metabolism disorder^[4,36]^. These five identified hub genes were involved in the underlying cause of OA, making them novel potential therapeutic targets.

The exposure of extracellular matrix and activation of innate immunity resulting from micro-articular damage have been well known as key factors in the progression of OA^[55]^. Initial joint injury triggers an inflammation cascade, worsening synovitis and contributing to OA advancement^[61]^. Currently, it is widely acknowledged that chronic intra-articular inflammation in OA is fueled by the activation of the body’s innate immune system, specifically due to an imbalance in immune cells such as macrophage polarization^[62]^ and B cell activation^[32]^. Recent evidence highlights the association between arthritic inflammation and adaptive immunity disorder, including cytokine imbalances and abnormal infiltration of B cells in OA^[63]^. While numerous studies have reported the aberrant enrichment and activation of B cells within the synovial tissue of OA joints^[27,32,33]^, the precise underlying mechanisms remain elusive. In the context of the knee joint, activated B cells are known to mutually enhance local inflammation response via the ERK1/2/JAK-STAT1 pathway^[64]^ and to promote fibroblast activation and migration through inflammatory mediators^[65]^. Collectively, these observations suggest a positive feedback loop between microenvironment and B cells within joint, potentially exacerbating disease progression. Our current study specifically identifies the C1 subpopulation in KOA joint tissue as a terminal differentiation stage of B cells. Notably, this subpopulation predominantly originates from elderly KOA patients, and its functional clustering primarily highlights B cell activation pathways. These findings underscore the critical need to focus on B cell activation, particularly in elderly KOA patients. Moreover, our results suggest significant associations between the transcription level of OA-related blood markers and the landscape of adaptive immunity (such as B and T cells) in the peripheral blood of OA patients. Additionally, the clinical phenotypes that are most related to older patients of OA were found to be significantly associated with the immune system (including innate and adaptive immune systems) in peripheral blood. While aging is known to decrease B cell activation and differentiation^[37,38]^, the flow cytometry analysis conducted in this study showed a higher proportion of total B cells and a more mature B cell phenotype in the older age group. Interestingly, there were no significant differences in late B cell differentiation levels, such as plasma cell (CD38^+^CD138^+^), memory B cell (CD19^+^CD27^+^LgM^−^), and regular B cell (CD19^+^CD24^+^CD38^+^). This observation suggests that the dysregulation of B cells in OA may overshadow the effects of age on B cells, and the absence of significant changes in memory or regulatory B cells implies that age-related immune dysfunction in OA may predominantly involve early activation phases rather than terminal differentiation. Thus, the disorder of the immune microenvironment and the imbalance of inflammatory factors in peripheral blood may play a leading role in OA, especially in older patients^[6,8,54]^.

Our study makes several important advances over previous OA research. First, while earlier studies have identified diagnostic genes in one or two joint components, our work is the first to systematically identify and validate five peripheral blood biomarkers (MAPK1, MAP3K8, ING1, LDLR, and NUP153) that are differentially expressed across all four major joint tissues (meniscus, cartilage, synovium, and subchondral bone) and peripheral blood. This comprehensive approach enhances the robustness and clinical relevance of our biomarker panel. Second, we demonstrate that these blood biomarkers exhibit age-specific expression patterns, and our refined diagnostic model shows superior predictive performance for elderly OA patients, addressing a critical gap in early detection for this high-risk group. Third, by integrating transcriptomics, single-cell RNA sequencing (scRNA-seq), and flow cytometry, we provide novel mechanistic insights into the immune landscape of OA, particularly highlighting aging-driven B cell remodeling and its contribution to disease progression. These findings not only advance the understanding of OA pathogenesis but also offer new avenues for non-invasive diagnosis and targeted immunomodulatory therapies, especially for the elderly population.

The present study has certain limitations. One of the primary limitations of this study is the small sample size between the OA and control groups. The limited number of cases may reduce the generalizability and statistical power of our findings. This constraint could potentially affect the robustness of the identified biomarkers and their association with OA. Additionally, the use of AI-based machine learning algorithms (random forest, SVM-RFE, and multivariate regression) for biomarker identification, while powerful, introduces potential limitations. These include the risk of overfitting, particularly with smaller datasets, which could lead to overly optimistic model performance. Future studies with larger sample sizes across diverse cohorts are necessary to validate our results and strengthen the conclusions drawn from this research. Although our findings indicated some blood biomarkers that could potentially aid in diagnosing OA and interact with peripheral blood immunity, While our study identifies and validates a set of peripheral blood biomarkers for OA, further functional studies, such as knockdown or overexpression of these genes in B cells, are necessary to establish direct causality and to better understand their roles in B cell dysfunction and OA pathogenesis Thus, further clinical and animal studies are needed to validate these results.

## Conclusions

Our study delineates a comprehensive framework bridging diagnostic biomarker discovery with age-imprinted immune dysregulation in OA. By integrating multi-tissue transcriptomic analyses, we identified five peripheral blood biomarkers (MAPK1, MAP3K8, ING1, LDLR, and NUP153) that robustly distinguish OA patients from controls, achieving exceptional diagnostic accuracy (AUC = 0.966). This model not only addresses the unmet need for non-invasive OA diagnostics but also underscores the interplay between molecular signatures and immune pathophysiology. Notably, we uncovered a striking age-dependent immune imbalance in OA, characterized by heightened B cell activation and differentiation in elderly patients. WGCNA and single-cell trajectory analyses revealed that B cells in OA joints follow distinct differentiation paths linked to antiviral responses and proliferative states. These findings were corroborated by flow cytometry, which demonstrated elevated CD24^+^ and IgM^−^ B cell subsets in elderly OA patients – a phenotype suggestive of immunosenescence or chronic antigenic stimulation.

## Data Availability

All AI outputs were reviewed and validated by the corresponding and senior authors. The data used and/or analyzed during the current study are available from the corresponding authors upon reasonable request.

## Sources of founding

This study was supported by grants from the Hefei Municipal Natural Science Foundation (HZR2444), the Key Project of Health Commission Applied Medical Research of Hefei (Hwk2024zd001), the Basic and Clinical Collaborative Research Promotion Initiative of the Third Affiliated Hospital of Anhui Medical University (2022sfy007, 2023sfy017), and the Anhui Medical University Foundation (2023xkj109).

## Research registration unique identifying number (UIN)

This study was registered with the Chinese Clinical Trial Registry (https://www.chictr.org.cn/, ChiCTR2400094845).

## Declaration of figures’ authenticity

All submitted figures are original creations by the author, who affirms that these images have not been reproduced and have not been previously published in whole or in part.
